# COVID-19 Pandemic and SMEs Performance Decline: The Mediating Role of Management Innovation and Organizational Resilience

**DOI:** 10.3389/fpubh.2022.944742

**Published:** 2022-07-12

**Authors:** Yunjian Li, Hongchuan Chen, Lulu Wei, Luqing Wei

**Affiliations:** School of Management, Guangzhou University, Guangzhou, China

**Keywords:** COVID-19 pandemic, performance decline, management innovation, organizational resilience, sudden crisis, SMEs

## Abstract

It is a major practical problem to find out a pathway for firms to quickly recover from the performance decline in the context of the COVID-19 pandemic and other sudden major crisis in the current academic circles. Based on event system theory and structural adjustment to regain fit model, this paper empirically explores the impact of the COVID-19 pandemic on SMEs performance decline and discusses the management innovation response and organizational resilience mechanism of firms by virtue of the questionnaire survey data of SMEs in Guangdong Science and Technology Park in China. The research results elucidate that the criticality and disruption of the COVID-19 pandemic will not only lead to the SMEs performance decline, but also enable SMEs to carry out management innovation. Moreover, management innovation does not directly curb the SMEs performance decline caused by the COVID-19 pandemic, but indirectly inhibit it by promoting organizational resilience. In other words, the COVID-19 pandemic will indirectly promote organizational resilience through firm management innovation, thereby curbing the SMEs performance decline. A path of management innovation response and organizational resilience to reverse the performance decline can be obtained in the study when SMEs confronting sudden major crisis. Furthermore, the study also expands the application scope of structural adjustment to regain fit model, which provides a useful reference for firm crisis response and sustainable development.

## Introduction

The COVID-19 pandemic (COVID-19) has forced a global blockade and economic shutdown (1), with a significant negative impact on production, operations and sales of firms, resulting in firm performance decline (*FPD*) (2). Against this background, many a firm all over the world are stuck deeply into the quagmire of the COVID-19 with great crisis and difficulties, and small and medium-sized enterprises (SMEs) were hit especially hard (3), may put SMEs' lives at risk (4), yet quite a few Chinese firms are still able to quickly revitalize from it. Therefore, it is of far-reaching practical significance to analyze the path of rapid resilience of the declining performance of Chinese firms under the background of the COVID-19, which will promote the sustainable development of firms and the healthy development of national economy in the sudden major crisis.

The existing research on the relationship between the COVID-19 and firm performance (*FP*) is mainly based on the analysis of corporate financial data, finding out the COVID-19 has a negative impact on *FP* (4–9) and firm sustainable growth (10). Nevertheless, there is a lack of empirical research on *SMEs*. In addition, some scholars have found that organization redundancy weakens the negative relationship between the COVID-19 and *FP* (5), and R&D investment plays a moderating role in the relationship between the COVID-19 and *FP* (8), customer concentration can relieve the negative impact of the COVID-19 on firm sustainable growth (10). The above studies mainly discuss the moderating function of redundant resources, R&D resources and customer resources from the perspective of resources, but not the mediating mechanism of the COVID-19 affecting *FP*. The existing research has ignored the theoretical premise that the resources can be used effectively to alleviate the chilling impact of the COVID-19 on *FP*. In the early period of the COVID-19, most firms come into a standstill, which brings about the shortage of these resources owned by firms and the difficulty to use them effectively. Under the circumstances, how to get these resources effectively used and back on track has become the top priority as to firms. However, there is short of discussion and research on how to deal with firm management innovation (*FMI*), how to achieve organizational resilience (*OR*) and performance improvement upon the major crisis.

The COVID-19 makes the external market environment more turbulent, which leads to the mismatch between the original organizational structure and the new environment. The mismatch between organizational structure and contingency factors causes a decline in performance, while the matching of the two factors will lead to the increase of performance (11). According to structural adjustment to regain fit model (SARFIT model) proposed by Donaldson (11), the change of contingency factors will lead to the adaptive change of organizational structure. Firms adopt new organizational structure to match the new contingency factors, contributing to the resilience of *FP*. Firms have to confront severe economic situation due to the drastic change of firm external environment caused by the COVID-19. Grim economic situation will prompt managers to regard innovation as the right strategy and adjust their management model to the crisis by virtue of a series of management innovation (*MI*) practices (12). *MI* is the invention and implementation of a new management practice, new process, new structure, or new technology to achieve further organizational goals (13). The practice of *MI* under crisis is the adaptive change of firms, and it is the process that firms adjust the mismatch between organizational structure and environmental factors to rematch. As a new practice beneficial to sustainability and performance improvement (14), *MI* offers a solution for companies breaking out of the COVID-19 dilemma and achieving resilience. *MI* is an effective measure in that it not only improves the economic benefit of firms in the short term, but also enhances the competitiveness and development potential in the long term. However, the abrupt outbreak of the COVID-19 has also changed the background of *FMI*. The conventional *MI* that the firm takes the initiative and carries on the *MI* step by step becomes the passive and emergency management innovation (*EMI*) that the firm carries on suddenly. What's worse, when implementing organizational changes such as new work organization or new knowledge management system, staff need a process of adaptation and learning, which means *MI* does not immediately transform into substantial improvements in innovation performance (15). In such a case of COVID-19, can *EMI* soothe *FPD*? If so, how does it reverse the trend of *FPD*?

With the COVID-19 and other major crisis, the premise of firms meliorating the *FPD* is to resume normal operations. The current business environment is characterized by a high degree of competition, uncertainty and ambiguity. Natural disasters and crisis occur frequently. These changes of the external environment magnify the importance of resilience to the organization (16). Organization resilience (*OR*) means an organization's ability to overcome difficulties, create opportunities and build a successful future by integrating resources from all sides. Resilience helps companies recover faster in the case of interrupted supply chain. The higher the resilience is, the better performance in terms of delivery performance, cost reduction and recovery situation they would be (17). Therefore, this study suggests that *OR* may be an important pathway for firms to reverse the trend of *FPD* caused by the COVID-19. Based on it, this study intends to further explore the following issues: Can *EMI* help firms achieve *OR* with the COVID-19, and then reverse the *FPD* caused by the COVID-19?

Based on the above considerations, this study intends to integrate the event system theory and the SARFIT model, constructing a *MI* response and *OR* mechanism model to deal with *FPD* caused by the COVID-19. Moreover, an empirical analysis is carried out by using the questionnaire survey data of firms in Guangdong Science and Technology Park in China. First and foremost, the study explores the impact of the COVID-19 on *FPD* and *MI*. Secondly, it discusses the intermediary conduction mechanism of *MI* and *OR* between the COVID-19 and *FPD*. At last, it pores over the mediating role of *MI* in changing *FPD* through *OR*, which provides some practical enlightenment for firms to carry out *MI* so as to promote *OR* and reverse the tide of *FPD* under the COVID-19 and other crises. The research contributions of this thesis are as follows.

Firstly, this study constructs and validates the input-process-output (*IPO*) mechanism model of firms' *OR* in the context of sudden and major emergency crisis, finding out the path of *MI* response and *OR* when firms want to reverse the *FPD*. At present, there are quite a few research on the application of event system theory. Most of them are qualitative and case studies, while quantitative empirical studies are relatively rare. This paper tries to put forward a theoretical framework of *IPO* about the impact of the COVID-19 on *MI, OR* and *FPD*, elaborating the criticality and disruption of the pandemic can promote the *OR*, and finally reverse the trend of *FPD* by enhancing *FMI*. From the perspective of event system theory, this paper reveals the intermediary conduction mechanism of the COVID-19 affecting *FP*, clarifies the path of *MI* and *OR* for firms to deal with sudden major crisis, and enriches the empirical research results of the event system theory as well.

Secondly, this paper extends Donaldson's (11) SARFIT model and finds the double-edged sword effect of the COVID-19. Based on the SARFIT model, this study tries to explain the process of *FPD* and *OR* caused by the COVID-19. It means that the COVID-19 incurs the imbalance between firm organizational structure and external environment, and then *FP* will fall off. While through the adaptive change of *MI*, firms can make them rematch to obtain the OR, which can reverse the *FPD* in return. In other words, although the COVID-19 will cause *FPD*, it can also prompt firms to carry on *MI*, then helping them to recover and revitalize the performance. It means that the COVID-19 has a double-edged sword effect. The research findings not only provide theoretical inspiration for firms to turn crisis into opportunity, but also apply the SARFIT model to the research field of firm crisis management, elucidating the phenomenon that firms implement *MI* and obtain *OR* with the sudden major crisis.

Thirdly, this paper digs out that *EMI* will result in the decline of short-term performance, and makes it clear that *OR* plays a mediating role between *MI* and *FPD*. Different from previous studies which have found that *MI* exerts a positive influence over *FP*, this study discovers that *EMI* has a positive effect on *FPD* under the impact of the COVID-19, and it will lead to a *FPD* in short-term. Maybe that's because *EMI* can cause short-term maladjustment, thus it can't bring about immediate and substantial melioration of *FPD*. In the sudden crisis, *MI* can't directly reverse the *FPD*. Only by virtue of *OR* can help firms restore from the *FPD* under the influence of the COVID-19. The findings generate a new insight for the role of *MI*, favorable to further understanding the theoretical black box between *MI* and *FP*, making up the deficiency of the existing research on the relationship between *MI* and *OR*, and enriching the research findings in the related fields of *MI* and *OR*.

The following contents of this article are as follows: the second part is literature review and research hypothesis; the third part is research design, including sample selection and variable description; the fourth part is empirical results and analysis, including reliability and validity test, descriptive statistics, correlation analysis, regression analysis and hypothesis test, and robustness test; The fifth part is the conclusions. The sixth part is the limitation and future research.

## Literature Review and Research Hypothesis

### Literature Review

#### Sudden Crisis and FP

The COVID-19 and other sudden crises will change the external environment on which firms depend and then affect the *FP*. According to literature review, it indicates that some scholars believe that crisis will have a negative impact on *FP*, yet such an influence can be adjusted by relevant factors. Kestens et al. (18) find that the financial crisis show a negative impact on *FP*, and trade credit could adjust the impact of the crisis on it. Ryu et al. (19) discover that natural disasters have a negative effect on organization performance, which could be mitigated by collaborative networks established in response to natural disasters. Li (5) finds that the COVID-19 has a negative impact on the performance of manufacturing firms, but organization redundancy weakens the negative relationship between the COVID-19 and *FP*.

On the other hand, some scholars hold a different view that crisis and *FP* are uncorrelated or positive correlation. De Freitas et al. (20) discover that there is no correlation between *FP* and other economic crisis. Some scholars believe that the sudden crisis not only bring threats, but also opportunities to firms. For instance, Noth et al. (21) declare that natural disasters have a positive effect on the *FP* since those firms have higher turnover, lower leverage ratio and higher cash flow after undergoing natural disasters.

#### Sudden Crisis and Firm Response

The abrupt outbreak of financial crisis, natural disasters, public hygiene and other crisis often makes the normal production and operation activities of firms affected or even interrupted. In the turbulent crisis environment, firms will take corresponding measures to adjust their own behavior to alleviate the adverse effects. Previous studies on firm crisis response have mainly focused on crisis interventions, including short-term emergency measures for survival and long-term strategic measures for development. The short-term emergency measures commonly used by firms are mainly such ways of maintaining business liquidity, developing short-term business, increasing income and reducing expenditure (22). Micro, small, and medium-sized firms will adjust workforce use by reducing working hours, arranging alternative jobs and layoffs, and take emergent measures to tackle the economic crisis such as exploiting new customers and markets, reducing costs and production (23). Since the outbreak of the COVID-19, the quarantine policies have restricted the mobility of people, and online telecommuting has become the most common-used measure for firms to deal with the pandemic (24, 25). While off-the-shelf information and communication technologies such as video conferencing can solve physical distance problems in the short and medium term, more advanced information and communication technologies such as virtual reality are more likely to become critical in the long term. The utility of information and communication technology has made business model innovation become a strategy of crisis response for firms (26). Among them, temporary business model adaptation is a short-term emergency measure, while continuous business model adaptation and identification of new value propositions are more in favor of long-term strategy (22).

In addition to getting through the crisis, firms entail a long-term strategic response to turn the crisis into an opportunity if they want to be stronger during crisis. Martin-rios et al. (27) find that service innovation is a long-term strategic adaptation activity adopted by top service firms in the EU during the economic crisis, among which increasing R&D investment, strategic mergers and acquisitions, and recruitment expansion can help firms to maximize the adaptation to crisis, ensure the long-term viability of strategic orientation, and promote the growth of operating profit, sales and market capitalization. It is true that innovation like digital innovation is a significant strategic response to crisis and long-term survival (28). Workflow digitization is an effective way for firms to overcome their long-term crisis (22). Digitization helps SMEs to cope with public crisis better and improve their performance as well (29). In addition, Martinelli et al. (30) point out that dynamic capabilities that firms reconstruct and utilize resources, perceive and interpret the environment, and learn to integrate knowledge can contribute to improving *OR* of firms in response to natural disasters, among which the ability of resource utilization is more suitable for short-term response, while the ability of resource reconstruction, environmental perception and interpretation are more favorable to long-term action. *OR* is beneficial to help firms turn crisis into safety with the environment of volatility, uncertainty, complexity, and ambiguity (*VUCA*).

### Research Hypothesis

#### The COVID-19 and FPD

*FPD* mainly shows that the actual performance of firms is worse than the expected performance. The expected performance level can be determined as the historical expected performance level based on the historical performance created by the firm capabilities and resources in the course of firm development, or based on the performance produced by other firms in the same industry to define the expected performance level of the industry. The *FPD* will be influenced by many a factor, such as manager's ability, firm resources, external environment and so on. The more uncertain factors they are, the more obvious impact on the *FPD* it will be. When emergency occurs, the entity usually does not prepare an effective response mechanism or procedure (31), so more or less it will cast a bad impact on the entity. The COVID-19 first occurred globally in January 2020 and spread quickly into a global pandemic (32). From the point of view of event system theory, the sudden outbreak of the COVID-19 has the characteristics of event's strength including novelty, disruption and criticality and so on, which will exert a series of influence on firm entities.

During the COVID-19, in order to prevent and control cross-infection and spread, government departments implement measures such as public health pandemic prevention, personnel isolation and travel restrictions, resulting in a sharp decline in population mobility, causing the direct blockade in terms of people, logistics and commodity flow (33). Due to it most people are isolated at home, and the out-of-home consumption activities plummet sharply, which reduce the consumption and demand of products and services, causing the original inventory backlog of firms, the increase of the inventory cost, and the reduction of firm profits. As a result, *FP* becomes lower than the historical level of the same period. At the same time, self-segregation and travel restrictions have less necessity for workforce in the economy sectors and lead to a sharp drop in jobs (34). Therefore, it increases the proportion of unemployed people, directly reduces the income of consumers, weakens the consumption power, and causes the *FPD* accordingly. The stagnant global stock markets is also attributive to the COVID-19 (35), triggering a signal to consumers that the expected business environment is unfavorable, and the uncertainty of consumers' expected income will increase. Such a situation in return affects consumers' confidence, expectations and behaviors, resulting in insufficient consumer demand, which in return causes *FPD*. The manpower tightness, the shortage of capital and supply chain disruptions caused by the COVID-19 are also the important reasons for it. During the COVID-19, most of the employees are isolated at home, and the firms are unable to resume work and production. Even if those firms that may resume work are also faced with the difficulties of insufficient manpower. And internal business activities fail to be carried out normally so that firms can't meet consumers' demand for products, which leads to *FPD*. Research shows that after the outbreak of crisis, people are inclined to avoid investment and remedy losses by withdrawing investment (36), and the decrease or even interruption of the supply of investment funds will have a negative impact on *FP*. At the same time, in order to avoid the cross-regional spread of the virus during the COVID-19, the geographical borders are blocked, which limits the services area of the logistics and transportation industries, letting firms fall into the plight of insufficient supply of raw materials, reducing the production capacity of the firm, and then leading to *FPD*. Moreover, large-scale shutdown has delayed the delivery of upstream production companies. Compared to large enterprises, SMEs have shorter life, lack preparedness when dealing with unforeseen circumstances (37), lack sufficient cash flow and are more vulnerable to labor shortage, and are affected negatively by the COVID-19 (38), which will lead to *FPD*.

Based on the above analysis, this paper proposes the hypothesis 1: the COVID-19 plays a significant positive role in SMEs *FPD*.

#### The COVID-19 and FMI

As an organism with self-healing function, firms will try to fit in with the changes by innovation when it is impacted by crisis. Existing research shows that SMEs will take measures to deal with the COVID-19, for example, transferring to the virtual space to make employees continue working remotely (38), implementing organization structure innovation (4), and other *MI* measures. *MI* is a new process in which firms bring about a shift in organization strategies, structures, procedures and systems by applying new knowledge and management methods (39). It is an adaptive change made by firms to respond to environmental changes. Environmental factor turns out to be a crucial point for *FMI* (40). Those managers who can perceive the external environment's change better is more sagacious to recognize new problems in time, so as to urge the firms to carry on the *MI* under crisis.

According to event system theory, those events not created by entities, but entailing entities to deal with passively are called passive events. The COVID-19 belongs to a passive event, which has the characteristics of novelty, disruption and criticality, leading to the mismatch between firms and new external environment, and forcing firms to put forward a series of *MI* measures. At first, the novelty of the COVID-19 that it is significant departure from previous public health with its sudden outbreak, high speed and wide range of spread, handling difficulty and long duration. As for the sudden outbreak of the COVID-19, firms lack corresponding management experience for reference, so they need to reformulate new solution to adapt to the current environment. Thereupon, firms will change internal communication methods, such as communication between employees and departments from offline to online, firm meetings from face-to-face communication to cloud communication like video conference. Companies will also shift their work patterns, moving flexible work arrangements online. More Internet-based remote work and less reliance on personnel gathering do good to maintaining social distancing for employees (41) and helping companies better fit into the outbreak. Secondly, the disruption of the COVID-19 is reflected in the hindrance and disruption of conventional entities activities. The pandemic spread more or less has impact on the economy, including trade reduction, supply shortages, and financial tightening. To alleviate the economic losses caused by the COVID-19, firms will change the traditional mode of offline sales in the entities to online sales model such as video live selling goods, short video marketing, and recommendation by WeChat official accounts. To adapt to the new sales model, firms will change their organization structure, such as the establishment of network marketing department, e-commerce department, and adjust the staff functions in time. Moreover, the criticality of the COVID-19 shows that firms need to give high priority to the pandemic. Since the outbreak of the COVID-19, firms has always put high premium on pandemic prevention and control to reorganize production patterns and take staggered shifts and so on. In addition to maintaining existing operations to ensure business liquidity, companies also explore long-term strategic changes to guarantee firms' viability (20). It shows that the COVID-19 will have a far-reaching impact on firms, may resulting in the mismatch of firms' original structure and external situation. Compared to large enterprises, SMEs are largely affected by external environmental changes (42). To accommodate to the changes in the external environment, SMEs will more likely to combine with the actual situation to achieve *MI* in the aspects of production process, operation modes, organization structures, task and function, management system, etc.

Based on the above analysis, this paper proposes the hypothesis 2: the COVID-19 has a significant positive effect on SMEs *FMI*.

#### MI and OR

During the pandemic period, due to home quarantine and other pandemic prion measures, it is difficult for firms to carry out business activities as usual, resulting in a mismatch between the original firm organization structure and the new external environment. *MI* stimulates the new knowledge that can change the existing capabilities of firms, making it more adaptable to the changing environment (43), and helping the rematch. *OR*, as a flexible dynamic capability that enables firms to adapt to and recover from unexpected adverse circumstances (44), represents the resilience of firms after its rematch. *OR* includes agility, integrity, and robustness, in which robustness reflects an organization's ability to withstand and recover from adverse conditions, agility measures an organization's ability to act quickly, and integrity reflects the cohesion of an organization's members in the face of adverse circumstances (16).

Innovation is the key factor to promote *OR* in sudden major crisis. Existing studies have found that the COVID-19 causes serious job insecurity and financial worries, affects the mental health of employees, and leads to negative emotions such as anxiety and depression (45). Anxiety, on the other hand, will affect an employee's work efficiency and target progress (46). Firms carry out *MI* by adjusting the task functions and salary structure of employees and using the new way of telecommuting or online and offline combination, which can help employees to adapt to the pandemic better and faster, reduce their negative emotions and economic worries, enhance their adaptability, improve individual resilience and then promote *OR*. The positive emotion of the staff is transmitted in the team through interactive communication, which facilitates collective positive emotional convergence of the team members and helps build team resilience (47). With positive emotions, team members show high cooperative willingness and team cohesion, which can help alleviate the damage and shock on team integrity caused by the COVID-19, thus enhancing team resilience and thus promoting *OR*. During the COVID-19, traditional management systems, work rules and procedures of firms are no longer fully applicable due to the restriction of pandemic prevention policies. Against this background, the establishment of new management systems and optimization of business procedures can enhance firms' adaptability and flexibility in the face of complicated environment, helping withstand risks and promote the robustness of firms. In addition, by means of information and communication technologies to adjust internal and external communication of firms not only facilitates effective communication and collaboration at all levels within firm, but also helps to maintain external social networking relationships, promote linkages between firms and stakeholders and enhance the robustness of their social networks. Furthermore, the flexibility provided by information and communications technology enables firms to rebuild and expand communication networks during disasters, collect resources and establish trans-geospatial working procedures. In this way may it promote *OR* of firms (48). Compared to large enterprises, SMEs are more flexibility. Therefore, when SMEs carry out *MI*, it is easier for them to recover to the normal operating level and obtain *OR*.

Based on the above analysis, this paper proposes hypothesis 3: SMEs *MI* has a significant positive effect on *OR*.

#### MI, OR and FPD

Existing research shows that the innovation practices adopted by SMEs to face the repercussions of COVID-19 had a positive impact on the performance (4). According to the SARFIT model of Donaldson (11), which has been successfully implemented in multiunit firms' research when subsidiary performance is below aspirations, and found that the subsidiary performance problems trigger structural adaptation in the internal governance mechanisms in pursuit of regaining fit (49). The structural adaptation has a positive impact on the performance of manufacturing SMEs (50). These studies show that the refitting between organizational structure and contingent factors can improve *FP*. The sudden outbreak of the COVID-19 makes firms face the dilemma of production and work stoppage, which leads to the mismatch between the organizational structure and the external environment. In this situation, firms will adjust their structure through *MI* to adapt to the change of contingency factors in order to regain the match, and then maintain the effective operation of firms and improve *FP*. For example, some Chinese enterprises, such as GREE, have taken actions to the COVID-19, which helps GREE reverse to the *FPD*. In 2020, the first quarter after the COVID-19, GREE's sales revenue decreased sharply by 49.01% year-on-year. In this context, GREE has developed strict and comprehensive prevention and control measures, established an epidemic prevention and control team, used online meetings whenever possible, stipulated that employees are not allowed to gather. Due to the shortage of workers, GREE requires all administrative personnel to enter the production line. Besides, GREE required, trained and guided all offline stores to start live selling, and adopted the management and assessment method of combining offline and online sales for the sales stores. Based on the above *MI* measures and sales organization structure adjustment, in the first half of 2020, GREE's sales revenue was decreased by 28.21% year-on-year, which means that the *FPD* has been reversed. This shows that enterprises' *MI* to deal with the COVID-19 may help to reverse *FPD*.

*MI* promotes the internal management ability of firms by adjusting the management practice, improving the business process, changing the organizational structure and so on. It can help firms to maintain strategic agility, operational robustness and team integrity under crisis, thus promoting *OR*. The implementation of *MI* can facilitate organizational change, organizational renewal, adaptation and effectiveness (39), and motivate organizational knowledge creation, sharing and application. *Via* knowledge-sharing mechanisms to obtain critical information in the external environment, firms can effectively deploy contingency plans and organize resources to facilitate internal knowledge and information exchange, so that they can better adapt to the changes, enhance *OR* of firms to withstand crisis. Using information and communications technology to achieve remote connections with employees may create a mutual vision for employees to move forward with their leaders about goals, key issues and work progress, which facilitates internal communication and create a shared environment for them to work together from a distance (48). By the telecommuting approach, as well as the adjustment and incentives of salary policies, employees can continue to work in a flexible way and have a positive psychological state under the crisis with less finance and unemployment concerns. Individual resilience allows employees to cope more successfully and effectively with uncertainty, rapid change, and pressure (51), which in return helps firms resume operations well after a crisis. Employees can contribute to *OR* through their positive psychology and communication behaviors (52). A firm's *OR* is supported by the collective resilience of its members, which the firm can leverage to generate higher value (53), enabling firms to adapt their structures more rapidly to environmental change.

*OR* contributes significantly to firm survival, business continuity and performance improvement. Firstly, resilient firms that can take advantage of their agility and flexibility are able to change course anytime at a low cost in order to transcend survival and thrive in complex and uncertain situation (53). Firms with agility can quickly and effectively integrate internal and external resources, adapt to situation changes, enhance their crisis response capacity, and effectively reduce the loss of firm resources. Agility significantly affects the product innovation ability of firms, which is conductive to develop new products better and faster (54), and to seize market opportunities, thus gaining an edge of market and improving *FP*. Resilient firms are more likely to increase their economic investment in resource conservation and sustainable production (1). Meanwhile, they can guarantee employees' health and safety, maintain their rights continuously, and enable them to stick to their post despite the crisis, thereby keeping the original organizational functions of firms and ensuring business continuity and organizational system robustness. Systems with robustness can consistently perform their expected functions even with interruptions (55). With the original function, business activities can be carried out as usual, so as to alleviate the impact of the COVID-19 to firms, minimize economic losses. In addition, the stronger the *OR* it is, the more likely firms are to find support and guidance from its resources and social networks when needed (56). Valuable and unique resources like information, knowledge, cooperation, and loyalty are embedded in the relationships established between firms and its major stakeholders. These resource advantages help firms cope with external shocks and bounce back from unexpected crisis during periods of uncertainty and severe volatility (57), which can reverse the trend of *FPD*.

Based on the above analysis, this paper proposes the hypothesis 4: SMEs *MI* can reverse the *FPD* caused by the COVID-19 through *OR*.

**Figure 1:**
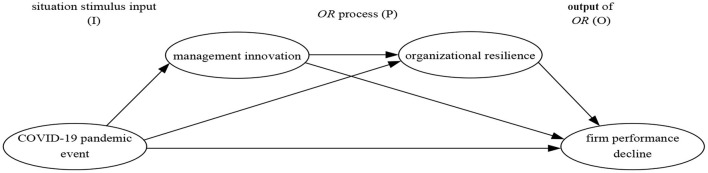
Research model.

To sum up, this paper regards that although the COVID-19 will lead to *FPD*, it will promote *MI* as well. *FMI* can spur *OR* and then reverse *FPD*. Therefore, this paper constructs the *IPO* mechanism of *FMI* response and *OR* under the sudden crisis, and regards the COVID-19 as the situation stimulus input (I), *FMI* response process as the *OR* process (P), and the improvement of performance as the output of *OR* (O. The research model of this paper is shown in Figure 1.

## Research Design

### Sample Selection

The object of this study is the SMEs from Guangdong Science and Technology Park in China. The survey period is from June 2020 to September 2020, and a total of 206 questionnaires have been collected, excluding five questionnaires with firms' established time over 20 years, 201 questionnaires left. The specific sample distribution is shown in Table 1. The sample firms mainly derive from information transmission, software and information technology services, scientific research and technology services. Most of them are SMEs new-established within 8 years with employees <300 and operating income <20 million yuan, and most of them have no overseas business. In a nutshell, the sample covers a wide range of industries with a good representation.

**Table 1:** Characteristics of valid samples.

**Variables**	**Categories**	**Frequency**	**Percentage**	**Variables**	**Categories**	**Frequency**	**Percentage**
Enterprise age	Within 8 years	168	83.582%	Industry attributes	Information transmission, software and information technology services	96	47.761%
	9–19 years	33	16.418%		Scientific research and technology services	43	21.393%
Number of employees	<20 employees	136	67.662%		Manufacturing	14	6.965%
	20–299 employees	63	31.343%		Leasing and business services	12	5.970%
	300–999 employees	2	0.995%		Culture, sports and entertainment	11	5.472%
Revenue of the previous year	Revenue < million	127	63.184%		Wholesale and retail	9	4.478%
	Revenue 3–20 million	59	29.353%		Other industries	16	7.960%
	Revenue 20–40 million	14	6.965%	Whether there is overseas business	Export	15	7.463%
	Revenue of more than 40 million	1	0.498%		No export	186	92.537%

### Variables Description

#### The Event's Strength of the COVID-19 Pandemic

The variable is based on the measuring instrument of Morgeson et al. (31), Morgeson (58) and Morgeson et al. (59) under the background of the COVID-19, including three dimensions: novelty, criticality and disruption. The novelty reflects in “there is a clear, known way for my company to respond to the COVID-19 pandemic event” and other three items. The criticality reflects in “the COVID-19 pandemic event is critical for the long-term success of my company's innovation and development” and other two items. The disruption reflects in “the COVID-19 pandemic event disrupts our company's value creation and acquisition ability to get its work done” and other three items. The variable is measured by Likert 5-point Scale, ranging from “1 = totally disagree” to “5 = totally agree”. The novelty is the reverse scoring question.

#### Management Innovation

This variable is mainly adapted from the measurement tool of Vaccaro et al. (60) and combined with the COVID-19 pandemic background. It includes that “rules and procedures within our organization are renewed during the COVID-19 pandemic” and other five items. The variable is measured by Likert 5-point Scale, ranging from ranging from “1 = totally disagree” to “5 = totally agree”.

#### Organizational Resilience

The variable is based on measurement tool of Kantur et al. (16) with the COVID-19 pandemic background, reflecting in “facing COVID-19 pandemic, our company is successful in generating diverse solutions,” and other eight items. The variable is measured by Likert 5-point Scale, ranging from “1 = totally disagree” to “5 = totally agree.”

#### Firm Performance Decline

This study measures the *FPD* from the dropping proportion of sales revenue, profit and market share, including “in the first half of 2020, the proportion of our company's sales revenue decline is expected to be” and other two items. The variable is measured by Likert 6-point Scale, namely “1 = no decline”, “2 = below 20%”, “3 = 20%~29%”, “4 = 30%~39%”, “5 = 40%~49%”, “6 = 50% and above.”

#### Control Variable

In this study, taking enterprise age, number of employees, revenue of the previous year, industry attributes, and whether there is overseas business as control variables.

In this study, the original measurement scale of the event's strength of the COVID-19, *MI* and *OR* are measured in English. This study adopts the back translation method to translate these English scales into Chinese and back into English. This study also adjusts the measurement items of the COVID-19 pandemic event's strength, *MI* and *OR* according to the COVID-19. Specific measurement items are shown in Table 2.

**Table 2:** Measurement items and the reliability and validity of variables (*N* = 201).

**Variable**	**Dimension**	**Item**	**EFA factor loading**	**CFA** **factor loading**	**Total variance** **explained**	**CR**	**Cronbach's α**
COVID-19 pandemic event's strength (KMO = 0.867)	Novelty	There is a clear, known way for our company to respond to the COVID-19 pandemic event®	0.901	0.915	30.574%	0.930	0.929
		There is an understandable sequence of steps that can be followed in responding for our company to the COVID-19 pandemic event®	0.921	0.943			
		Our company can rely on established procedures and practices in responding to the COVID-19 pandemic event®	0.898	0.834			
		Our company had rules, procedures, or guidelines to follow when the COVID-19 pandemic event occurred®	0.847	0.809			
	Criticality	The COVID-19 pandemic event is critical for the long-term success of our company's innovation and development	0.703	0.773	58.193%	0.880	0.875
		The COVID-19 pandemic event is of a priority to our company's innovation and development	0.846	0.846			
		COVID-19 pandemic is an important event for our company's innovation and development	0.783	0.903			
	Disruption	The COVID-19 pandemic event disrupts our company's value creation and acquisition ability to get its work done	0.765	0.772	78.686%	0.876	0.872
		The COVID-19 pandemic event causes our company to stop and think about how to respond	0.744	0.799			
		The COVID-19 pandemic event alters our company's normal way of responding	0.861	0.852			
		The COVID-19 pandemic event requires our company to change the way it does its work	0.835	0.773			
Management innovation (KMO = 0.865)		Rules and procedures within our organization are renewed during the COVID-19 pandemic	0.864	0.850	72.816%	0.925	0.922
		Our organization make changes to our employees' tasks and functions during the COVID-19 pandemic	0.863	0.840			
		Our organization implements new management systems during the COVID-19 pandemic	0.886	0.867			
		The policy with regard to salary has been changed during the COVID-19 pandemic	0.787	0.715			
		The intra-and inter-departmental communication structure within our organization is restructured during the COVID-19 pandemic	0.890	0.877			
		our organization continuously alter certain elements of the organizational structure during the COVID-19 pandemic	0.825	0.764			
Organizational Resilience (KMO = 0.878)		Facing COVID-19 pandemic, our company stands straight and preserves its position	0.725	0.679	58.625%	0.909	0.905
		Facing COVID-19 pandemic, our company is successful in generating diverse solutions	0.694	0.643			
		Facing COVID-19 pandemic, our company shows resistance to the end in order not to lose	0.548	0.517			
		Facing COVID-19 pandemic, our company does not give up and continues its path	0.816	0.795			
		Facing COVID-19 pandemic, our company rapidly takes action	0.833	0.830			
		Facing COVID-19 pandemic, our company develops alternatives in order to benefit from negative circumstances	0.756	0.692			
		Facing COVID-19 pandemic, our company is agile in taking required action when needed	0.874	0.856			
		Facing COVID-19 pandemic, our company is a place where all the employees engaged to do what is required from them	0.765	0.698			
		Facing COVID-19 pandemic, our company is successful in acting as a whole with all of its employees	0.830	0.784			
Firm performance decline (KMO = 0.698)		In the first half of 2020, the proportion of our company's sales revenue decline is expected to be	0.962	0.968	87.390%	0.933	0.927
		In the first half of 2020, the proportion of our company's profit decline is expected to be	0.964	0.983			
		In the first half of 2020, the proportion of our company's market share decline is expected to be	0.875	0.756			

## Empirical Results and Analysis

### Testing of Reliability and Validity and Common Method Bias

Considering that the core scales used in this study are all adapted based on the background of the COVID-19, the study uses exploratory factor analysis (EFA) in order to test the validity and reliability of the adapted scale. As shown in Table 2, the KMO value of the COVID-19 event's strength is 0.867, which indicates that the COVID-19 event's strength is suitable for EFA. By the maximum variance method three factors selected, the results show that the items are well clustered into three dimensions: novelty, criticality and disruption, and the total variance of cumulative interpretation is 78.686%, which indicates that the scale has good validity. The Cronbach's α values of novelty, criticality and disruption of the COVID-19 is 0.929, 0.875, 0.872 respectively, and the composite reliability values are 0.930, 0.880, 0.876 respectively, which shows that the scale has good internal consistency and reliability. The KMO value of the MI is 0.865, which indicates it is suitable for EFA. One factor is extracted by using the maximum variance method, and the total variance of cumulative interpretation is 72.816%, showing that the scale has good converge validity. As to MI, its Cronbach's α is 0.922 and its combined reliability is 0.925, which reflects that the scale has good internal consistency and reliability. The KMO value of OR variable is 0.878, which indicates that the scale is compatible to EFA. One factor is extracted by using the maximum variance method, and the total variance for the cumulative interpretation is 58.625%, evincing that the scale has good converge validity. The Cronbach's α of the OR variable is 0.905 and its composite reliability is 0.909, showing that the scale possesses good internal consistency and reliability. The KMO value of FPD is 0.698, congruent for EFA. Extracting one factor is through the maximum variance method, and the total variance for the cumulative interpretation is 87.390%, declaring a good converge validity. The Cronbach's α of FPD and its combination reliability are 0.927 and 0.933, respectively, which shows the scale has good internal consistency and reliability.

Based on exploratory factor analysis (EFA), confirmatory factor analysis (CFA) is performed to test the discrimination validity. The results are shown in Table 3. It can be seen that the fitting degree of six-factor model (χ^2^/*df* = 1.973 < 3, *RMSEA* = 0.070 < 0.08, *CFI, TLI, IFI* > 0.9, *PCFI* > 0.5) is superior to single-factor, two-factor, three-factor, four-factor and five-factor models, indicating that there is a good distinction between novelty, criticality, disruption of MI, OR and FPD. In addition, the factor loading of CFA ranges from 0.517 to 0.983, all of which are bigger than 0.5, ensuring a good validity. In terms of common method bias test, Harman's single-factor test method used firstly shows that factor 1 accounts for 18.821% of the variance, <40% and not exceeding half of the 70.899% of the total variation, which means the method is feasible. The results of EFA in Table 3 show that the fitting degree of single-factor model is very poor, which is much lower than that of six-factor model. And the changes of *RMSEA* and *CFI* are 0.002, 0.001 respectively after controlling common method factors, the variation is <0.05, which evinces that the common method deviation could be well controlled.

**Table 3:** Confirmatory factor analysis results (*N* = 201).

**Variables**	***χ^2^***	***df***	***χ^2^ /df***	***RMSEA***	***CFI***	***TLI***	***IFI***	***PCFI***
6-factor model	710.220	360	1.973	0.070	0.923	0.913	0.924	0.819
6-factor model + common method factor	684.644	337	2.032	0.072	0.924	0.908	0.925	0.767
5-factor model	1,256.175	365	3.442	0.110	0.804	0.782	0.806	0.723
4-factor model	1,477.050	371	3.981	0.122	0.757	0.734	0.759	0.692
3-factor model	2,161.381	374	5.779	0.155	0.608	0.574	0.610	0.560
2-factor model	2,810.093	376	7.474	0.180	0.466	0.423	0.469	0.431
1-factor model	3,248.710	377	8.617	0.195	0.370	0.321	0.374	0.343

*6-factor model: novelty, criticality, disruption, MI, OR, FPD. 5-factor model: novelty, criticality, disruption, MI + OR, FPD. 4-factor model: novelty + criticality + disruption, MI, OR, FPD. 3-factor model: novelty + criticality + disruption, MI+OR, FPD. 2-factor model: novelty + criticality + disruption, MI+OR+FPD. 1-factor model: novelty + criticality + disruption + MI + OR + FPD*.

### Descriptive Statistics

According to the *FPD* of various industries under the COVID-19, the COVID-19 has the greatest impact on the accommodation and catering, entertainment, education, water environment and public facilities management sectors. And it has a greater impact on agriculture, forestry, animal husbandry and fishery, manufacturing, wholesale and retail trade, and financial industry, while exerts less influence on health and social work, neighborhoods' services, repair and other services.

Specifically, Among the sample firms in the first half of 2020, there are 37 firms whose sales revenue year-on-year decrease is <20%, accounting for 18.4%; There are 17 firms whose sales revenue year-on-year decrease is 20%−29%, accounting for 23.4%. There are 47 firms whose sales revenue year-on-year decrease is between 40% and 9%, accounting for 8.5%; There are 34 firms whose sales revenue year-on-year decrease is above 50%, accounting for 16.9%. There are 41 firms without decline in sales revenue, accounting for 20.4%. In the first half of 2020, there are 40 firms whose profit year-on-year decrease is within 20%, accounting for 19.9%, and 49 firms' profit year-on-year decrease is between 20 and 29%, accounting for 24.4%. There are 25 firms whose profit year-on-year decrease is between 30 and 39%, accounting for 12.4%, and 17 firms whose profit decrease is from 40 to 49%, accounted for 8.5%. There are 33 firms whose sales revenue year-on-year decrease is 50% and above, accounting for 16.4%. There are 37 firms without decline in sales revenue, accounting for 18.4%. In the first half of 2020, there are 68 firms with their market share decrease is <20%, coming in at 33.8%. There are 45 firms whose market share year-on-year decrease is between 20 and 29%, accounting for 22.4%, and there are 27 firms whose market share year-on-year decrease is between 30 and 39%, accounting for 13.4%. There are 12 firms whose market share year-on-year decrease is between 40 to 49%, coming in at 6.0%, and there are 17 firms whose market share year-on-year decrease is 50% and above, accounting for 8.5%. The market share of 32 firms without decline, accounting for 15.9%. Overall, about 80% of the sample firms experience a decline in sales revenue, profit and market share, while more than 16% of firms have a decline year-on-year in their sales revenue and profits by more than 50%. It elucidates that under the influence of COVID-19, *FP* generally declines and even some firms have a significant decline in performance.

### Correlation Analysis

As the results of Table 4 shows, the novelty mean value of the COVID-19 is 1.901, which is much lower than the criticality (*m* = 3.731) and disruption (*m* = 3.586). It shows that the sample firms have the corresponding methods, procedures, rules and guidelines to deal with the COVID-19.

**Table 4:** Means, standard deviations and correlations coefficients (*N* = 201).

	**Mean**	**SD**	**1**	**2**	**3**	**4**	**5**	**6**
1 *novelty*	1.901	0.710	**0.877**					
2 *criticality*	3.731	0.795	−0.422^***^	**0.842**				
3 *disruption*	3.586	0.817	−0.234^***^	0.692^***^	**0.780**			
4 *MI*	3.611	0.754	−0.355^***^	0.443^***^	0.375^***^	**0.821**		
5 *OR*	3.774	0.608	−0.507^***^	0.286^***^	0.199^**^	0.435^***^	**0.728**	
6 *FPD*	3.093	1.516	0.026	0.350^***^	0.498^***^	0.265^***^	−0.065	**0.908**

*** *p < 0.001*.

** *p < 0.010*.

*p < 0.050.

*The bold part in the table is the AVE square root of each latent variable*.

The mean value of *FPD* of sample firms is 3.093, and the standard deviation is 1.516, which manifests that different firms have different *FPD*. The novelty is negatively correlated with *MI* and *OR*, but not significantly correlated with *FPD*. Criticality and disruption are positively correlated with *MI, OR* and *FPD*. The preliminary results show that the novelty of the COVID-19 does not promote the *FMI* and lead to *FPD*, while the criticality and the disruption of the will result in *FPD* but it will enhance *FMI* and then obtain *OR* at the same time.

### Regression Analysis and Hypothesis Test

The SPSS 22.0 software and Model 6 in the macro program process are applied for regression analysis (The regression results are shown in Table 5). From Model 1, Model 2 and Model 3, it can be concluded that the COVID-19 has a significant negative effect on business *MI* (β = −0.376, *p* < 0.001), and the criticality (β = 0.406, *p* < 0.001) and disruption (β = 0.327, *p* < 0.001) of that have positive effects on business *MI*. The results shows that the criticality and disruption of the COVID-19 could promote *FMI*, while the novelty of the COVID-19 could not. Therefore, the hypothesis 2 gets some support. Model 4, Model 5 and Model 6 shows that there is a significant negative correlation between the novelty of the COVID-19 and *OR* (β = −0.345, *p* < 0.001). Yet the regression coefficients of the criticality and disruption of the COVID-19 are not significant (β = 0.089, *p* > 0.050; β = 0.048, *p* > 0.050). However, in all the three models, *MI* has significant positive effect on *OR* (β = 0.247, *p* < 0.001; β = 0.323, *p* < 0.001; β = 0.346, *p* < 0.001), indicating that the event's strength of the COVID-19 does not directly promote *OR*, but indirectly by promoting *FMI*. Thus, the hypothesis 3 is supported. From Model 7, Model 8 and Model 9, it can be seen that the criticality and disruption of the COVID-19 have significant positive effect on *FPD (*β = 0.591, *p* < 0.001; β = 0.841, *p* < 0.001), but the regression coefficient of the novelty of the COVID-19 is not significant (β = 0.056, *p* > 0.050). The result shows that the criticality and disruption of the COVID-19 could lead to *FPD*, while the novelty of the COVID-19 does not. Hypothesis 1 gets some support.

**Table 5:** Results of hierarchical regression analyses (*N* = 201).

	***MI***			***OR***			***FPD***		
	**Model 1**	**Model 2**	**Model 3**	**Model 4**	**Model 5**	**Model 6**	**Model 7**	**Model 8**	**Model 9**
*Novelty*	−0.376^***^			−0.345^***^			0.056		
*Criticality*		0.406^***^			0.089			0.591^***^	
*Disruption*			0.327^***^			0.048			0.841^***^
*MI*				0.247^***^	0.323^***^	0.346^***^	0.714^***^	0.464^**^	0.411^**^
*OR*							−0.476^*^	−0.594^***^	−0.580^***^
*Enterprise age*	0.001	0.0001	0.003	−0.004	−0.0004	0.001	0.003	−0.007	−0.008
*Number of employees*	0.110	0.048	0.077	0.104	0.064	0.068	−0.0002	−0.012	0.028
*Revenue of the previous year*	−0.055	0.035	0.054	−0.031	0.029	0.029	−0.467^*^	−0.425^*^	−0.338
*Dummy variable_ Manufacturing*	−0.480	−0.346	−0.308	0.165	0.217	0.223	−0.428	−0.319	−0.072
*Dummy variable_ overseas*	0.411^*^	0.409^*^	0.309	−0.138	−0.167	−0.191	0.056	0.146	−0.096
Constant	4.256	1.976	2.243	3.463	2.146	2.214	2.893	2.129	1.287
*R*	0.415	0.479	0.412	0.585	0.465	0.457	0.397	0.482	0.571
*R* ^2^	0.173	0.229	0.170	0.342	0.216	0.209	0.158	0.232	0.326
*F*	6.742^***^	9.619^***^	6.597^***^	14.344^***^	7.604^***^	7.281^***^	4.499^***^	7.263^***^	11.631^***^

*** *p < 0.001*.

** *p < 0.010*.

* *p < 0.050*.

In addition, Model 7, Model 8 and Model 9 show a significant positive correlation between *MI* and *FPD* (β = 0.714, *p* < 0.001; β = 0.464, *p* < 0.010; β = 0.411, *p* < 0.010), which shows that *EMI* will also lead to the *FPD* when crisis come. However, *OR* has a significant negative effect on *FPD* (β = −0.467, *p* < 0.050; β = −0.594, *p* < 0.001; β = −0.580, *p* < 0.001), which means that *OR* can reverse the situation of *FPD*. In summary, *EMI* cannot directly improve *FPD*, but indirectly restrain *FPD* by promoting *OR* in the context of the COVID-19. Thus, the hypothesis 4 is supported.

### Robustness Test

**Figure 2:**
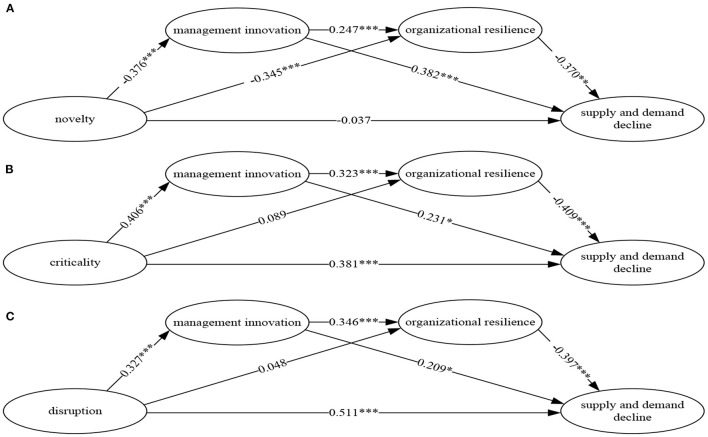
**(A–C)** Path coefficient of COVID-19 pandemic event's strength, *MI, OR* and supply and demand decline. ***P < 0.001, **P < 0.010, *P < 0.050.

A robustness test by using supply and demand recession as the variable. Considering that the decline of supply and demand means the decline of performance, this paper uses the supply and demand recession as the substitute variable for robustness test. The supply and demand recession of firms includes four items, the proportion of production capacity decline, order decline rate, the proportion of raw material gap, and employee turnover rate. (The variable, Cronbach's α is 0.845, and the factor loading of the items is 0.732–0.919. The cumulative explained total variance is 68.456%). On this basis, this paper examines the impact of the event's strength of the COVID-19 on *MI, OR*, and supply and demand recession. The results are shown in Figure 2. It shows that the result of regression test is consistent with that of *FPD* as a dependent variable, which shows that the result of this study has sound robustness.

Based on the above analysis, the research hypotheses 1 and 2 of are partially supported, and hypotheses 3 and 4 are supported.

## Conclusions

Based on the event system theory and SARFIT model, this study constructs a research model covering the COVID-19, *MI, OR* and *FPD* and performs a questionnaire survey among SMEs in Guangdong Science and Technology Park in China in the context of the COVID-19 to empirically explore how SMEs reverse *FPD* in sudden major crisis. Conclusions are as follows.

Firstly, the criticality and disruption of the COVID-19 will not only lead to *FPD*, but also encourage *FMI*. The results of the empirical study show that for the SMEs, the novelty of the COVID-19 is relatively low, and most of the sample firms have the corresponding methods, procedures, rules and guidelines to deal with the COVID-19. Moreover, the novelty of the COVID-19 doesn't cause *FPD*, and it is not conducive to *FMI* and *OR*. But the COVID-19 enjoys relatively high criticality and disruption to the SMEs, which explains that the COVID-19 has the important influence on SMEs, and they need to carry on innovation response. The empirical results further show that the criticality and disruption of the COVID-19 is positively correlated with *FPD* and *MI*, which indicates that the criticality and disruption of the COVID-19 will lead to *FPD*, and prompt SMEs to carry on *MI* response as well.

Secondly, the event's strength of the COVID-19 does not directly promote *OR*, but indirectly influences the *OR* by promoting *FMI*. The results of the empirical study show that the novelty of the COVID-19 has a negative effect on *OR*, while the criticality and disruption of the COVID-19 have no significant direct effect on *OR*. The results show that the event's strength of the COVID-19 could not directly promote *OR*. However, the event's strength of it will directly affect *FMI*. The novelty of the COVID-19 has a significant negative impact on *FMI*, while the criticality and disruption of the COVID-19 have a significant positive influence on the *MI*, which indicates that the former is not conducive to the *MI* and *OR* of firms, but the latter can prompt SMEs to carry out *MI* to indirectly promote *OR*. The results show that the different characteristics of the event's strength of the COVID-19 have different effects on *MI* and *OR*.

Thirdly, *MI* cannot directly curb *FPD* caused by the COVID-19, but indirectly reverse it by promoting *OR*. As empirical study shows that SMEs carrying out *EMI* in response to major crisis cannot directly restrain *FPD* caused by the COVID-19. On the contrary, the *EMI* is positively correlated with *FPD*, which indicates that *EMI* is not conducive to the improvement of *FP* in the short term. The results declare that *MI* has a significant positive effect on *OR*, while *OR* has a significant negative correlation with *FPD*. In other words, *OR* is the medium transmission mechanism of *MI* to reverse the trend of *FPD*. Only by *OR* can SMEs reverse the *FPD* caused by major crisis such as the COVID-19 to help the business return to normal development.

Based on the above conclusions, this paper puts forward the following suggestions. First and foremost, firms should critically treat major crisis such as the COVID-19 and learn to leap to safety. With current turbulent market environment, sudden crisis are inevitable, managers need to deal with *FPD* at any time, and consider how to achieve sustainable development. This paper finds that although unexpected crisis such as the COVID-19 may give rise to *FPD*, firms can carry out *MI* in response to crisis by exerting their own subjective initiative. In return, it can contribute to *OR* and curb *FPD*. Public emergent crisis like the COVID-19 is hard to predict and inevitable for firms, but it also provides them with the opportunity to reexamine the original organizational system. It can urge managers to improve their firm's current management systems, work procedures and so on, helping them accumulate crisis management experience. Therefore, firms should treat crisis correctly, draw lessons from crisis management, and learn to achieve *OR* to head off a danger through *MI* and other measures.

Secondly, firms should initially take *MI* and other actions to deal with the COVID-19 and other major emergencies. In such a market environment with *VUCU*, firms should take the initiative to effectively cope with crisis. Therefore, they can strengthen the application of digital technology, promote the firm management toward digitalization transformation, and enhance *MI* when firms respond to the sudden crisis. Meanwhile, they can use digital technology for employees to create a digital work platform, providing security for them to work in times of crisis, and to help firms maximize the normal operation. At the same time, they can strengthen the construction of digital infrastructure, improve the internal basic data interfaces for all types of business, open up and connect the corresponding subsystems for all types of business, and promote data sharing and business synergy, enhancing the system agility of firms. Finally, they can enhance firms' ability to predict crisis *via* digital technology to collect and analyze warning data, and establishing a timely response mechanism for crisis response plans so as to help firms meditate the harm of crisis.

Thirdly, firms should attach importance to enhancing *OR* through digitization and crisis management training. *OR* is the core of firms to deal with sudden crisis and reverse *FPD*. Firms should pay attention to building *OR* cultivation and promotion mechanism. Moreover, they can use digital technology to build data sharing platform for knowledge and information exchange, which can improve organization communication and work efficiency, and enhance the agility of individual employees and firms as a whole. Furthermore, companies can utilize social media to interact with stakeholders, establish partnerships with mutual benefit and reliance, and enhance the robustness of their networks. The last but not least, they should also value the training and the promotion of leadership crisis management capability, notice the staff and the teams' mental and physical pursue and enhances the staff's organizational commitment and the team cohesive force, strengthening the organizational integrity of firms when confronting crisis.

The COVID-19 is a global public health event, and it is still affecting enterprises in the world today. During the COVID-19, most countries adopted quarantine policies, for example, Japan, South Korea, Germany, Britain, the United States, Australia, etc. For enterprises in these countries and regions, they also suffered from the impact of the COVID-19, they also need to adopt *MI* to deal with the government's quarantine policies, and they also need to pay attention to improving their *OR* to deal with sudden major crises. Therefore, the implications on taking *MI* action and improving *OR* can also be used as a reference for enterprises in other countries.

## Limitations and Future Research

Although this paper has made some contributions, there are still some limitations. First, this paper is an empirical study based on SMEs in China. Like most local studies based on a national sample, the selection of research samples has limitations. Therefore, in the future, international research cooperation should be carried out, sample enterprises from more countries should be selected for comparative analysis, and comparative analysis of different types of enterprises should be carried out.

Second, because it is difficult to obtain the first-hand longitudinal survey data at the enterprise level, this paper has the limitation of a single data source. In the future research, it is better to select different sources to obtain multi-stage longitudinal research data, so as to further analyze the dynamic impact of major public health events such as COVID-19 on enterprises and the dynamic response strategies of enterprises, and then more accurately determine the causal relationship between major public health events, enterprises' innovation response and enterprise performance.

Third, how to recover from a major crisis, reverse the *FPD* and carry out sustainable development is a more complex process. With the continuous spread of the COVID-19, in order to cope with the impact of the epidemic, in addition to *MI*, enterprises will also carry out business model innovation and use digital technology for innovation and development. Therefore, future research should further analyze the impact of major public health events on enterprises in terms of the event's time and space, and comprehensively consider the impact mechanism of business model innovation, digital innovation and *MI* on firm performance.

## Data Availability Statement

The original contributions presented in the study are included in the article/supplementary material, further inquiries can be directed to the corresponding author.

## Ethics Statement

Ethical review and approval was not required for the study on human participants in accordance with the local legislation and institutional requirements. The participants provided their written informed consent to participate in this study.

## Author Contributions

YL and HC designed the study, collected the data, and revise the full text. YL wrote the introduction, performed the statistical analyses, discussed the results and wrote the conclusions. HC contributed to the implications. LulW and LuqW wrote the first draft of the literature review research hypothesis. All authors contributed to manuscript revision, read, and approved the submitted version.

## Funding

This research was supported by the National Natural Science Foundation of China (71802062), Science and Technology Planning Project of Guangdong (2020B1010010013 and 2018A070712042).

## Conflict of Interest

The authors declare that the research was conducted in the absence of any commercial or financial relationships that could be construed as a potential conflict of interest.

## Publisher's Note

All claims expressed in this article are solely those of the authors and do not necessarily represent those of their affiliated organizations, or those of the publisher, the editors and the reviewers. Any product that may be evaluated in this article, or claim that may be made by its manufacturer, is not guaranteed or endorsed by the publisher.
